# Relationship between hypoxia and response to antiangiogenic therapy in metastatic colorectal cancer

**DOI:** 10.18632/oncotarget.8712

**Published:** 2016-04-12

**Authors:** Paola Ulivi, Giorgia Marisi, Alessandro Passardi

**Affiliations:** ^1^ Biosciences Laboratory, Istituto Scientifico Romagnolo per lo Studio e la Cura dei Tumori (IRST) IRCCS, Meldola, Italy; ^2^ Department of Medical Oncology, Istituto Scientifico Romagnolo per lo Studio e la Cura dei Tumori (IRST) IRCCS, Meldola, Italy

**Keywords:** metastatic colorectal cancer, hypoxia, angiogenesis, bevacizumab, antiangiogenic therapy

## Abstract

Colorectal cancer remains a major public health problem worldwide. Despite the introduction of antiangiogenic drugs for the treatment of metastatic disease, a large number of issues remains unresolved. In particular, studies on predictive biomarkers of response and pathways of resistance to these agents are lacking, making it difficult to accurately select candidates for treatment. Hypoxia is the prime driving force for tumor angiogenesis and a vicious cycle between hypoxia and angiogenesis can be observed in tumors. Anti-angiogenic drugs act inhibiting tumor vasculature and, as consequence, inducing hypoxia. However, hypoxia could, in turn, induce an increase of metastatic potential of cells and a series of phenomena that could induce drug resistance. In the present review biological mechanisms of hypoxia and its relation with angiogenesis, and resistance to antiangiogenic therapy will be discussed. Moreover, data from clinical trials on antiangiogenic drugs in metastatic colorectal cancer will be reviewed, and the role of hypoxia in monitoring the response to treatment will be analysed. Combination strategies using anti-angiogenic and hypoxia inhibiting drugs are also discussed as they constitute promising field of research.

## INTRODUCTION

Colorectal cancer is one of the commonest cancers in the world [1]. Over the last decade, the development of targeted therapies has enriched the therapeutic armamentarium in the management of metastatic disease, resulting in significant gains in patient survival.

In particular, angiogenesis targeting *via* the vascular endothelial growth factor (VEGF) pathway has raised the attention of clinicians, considering the favourable survival benefit given by bevacizumab (Bev) in clinical trials in both first [2] and second line [3] settings. More recently, large phase III studies have shown clinical efficacy also in the new anti-angiogenic agents Ziv-aflibercept [4] and regorafenib [5]. However, benefits of angiogenesis inhibitors (AI) in an unselected patient population are modest. To date the research of predictive biomarkers has been unsuccessful and the mechanisms of resistance to such agents are unknown [6]. Induction of hypoxia represents a constant event during treatment with an antiangiogenic drug, and it is, in turn, a mechanism responsible for resistance to therapy.

In this review we examine the principal AI used in clinical practice for metastatic colorectal cancer (mCRC) and focus on the biological mechanisms of hypoxia, especially in relation to angiogenesis, which may be responsible for resistance to therapy. We also discuss how hypoxia could be used to monitor the response to these drugs, and argue the hypothesis for using combination strategies composed of AI and agents that are capable of inhibiting hypoxia.

## ANGIOGENESIS INHIBITORS IN COLORECTAL CANCER TREATMENT

**Figure 1:**
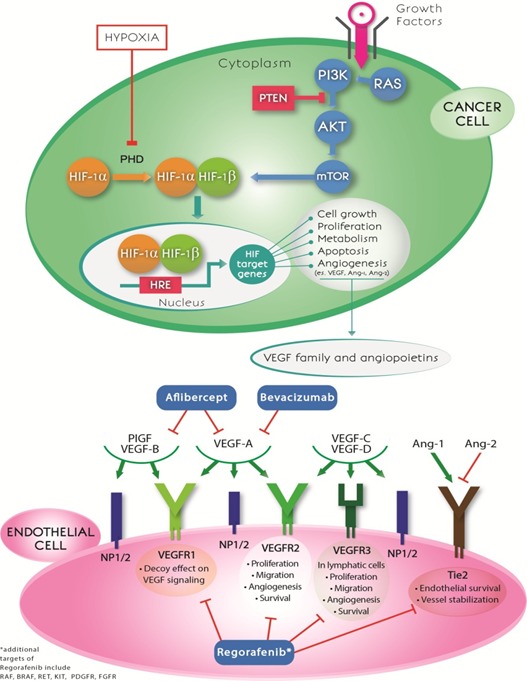
Anti-angiogenic drugs and crosstalk between hypoxia and angiogenesis pathways The mechanisms of action of the three anti-angiogenic drugs (bevacizumab, aflibercept and regorafenib) commonly used in mCRC treatment are shown in the lower part of the figure. Hypoxia inhibits the activity of PHD enzymes, allowing HIF-1α proteins to be stabilized, to dimerize with HIF-1β and to translocate to the nucleus. This complex binds hypoxia response elements (HREs) within the promoters of target genes. HIF-target genes are involved in cell growth and survival, proliferation, metabolic reprogramming, apoptosis and induction of angiogenesis mediated by vascular endothelial growth factor and angiopoietins. HIF-1α may also be upregulated in tumor cells by the activation of the PI3K-AKT-mTOR pathway. VEGF family members and angiopoietins interact with their receptors (VEGFR-1,-2,-3, NP1/2, Tie2), leading to different biological consequences. HIF: hypoxia-inducible factor; PHD: prolyl hydroxylase; HRE: hypoxia response element; PlGF: placenta growth factor; VEGF: vascular endothelial growth factor; VEGFR: vascular endothelial growth factor receptor; NP: neuropilin; Ang: angiopoietin.

Over the last decade 3 AI have been approved by the US Food and Drug Administration (FDA) for the treatment of mCRC: Bev in 2004, Ziv-aflibercept and Regorafenib in 2012. Bev is a humanized immunoglobulin (Ig) G1 monoclonal antibody directed against all isoforms of VEGF-A. It binds to VEGF-A inhibiting its link to the receptors (VEGFR-1,-2) on the surface of endothelial cells. As a consequence, the proliferation of endothelial cells and the creation of new blood vessels are blocked [7]. Ziv-Aflibercept is a fusion protein AI, designed to bind to VEGF-A, VEGF-B, and PIGF with higher affinity than their native receptors (VEGFR-1,-2). It acts as a “VEGF trap”, thus inhibiting multiple pathways involved in oncogenesis and tumor angiogenesis [8]. Regorafenib is a small molecule, multi-kinase inhibitor, acting against a wide range of tyrosine kinases including RET, VEGFR, KIT, PDGFR, FGFR, TIE2, DDR2, TrkA, Eph2A, RAF-1, BRAF, BRAFV600E, SAPK2, PTK5, and Abl [9] (Figure 1).

Data from the major phase III trials that have led to drug approval are being reviewed in this section.

Several phase III randomised clinical trials have investigated the efficacy of Bev added to first-line chemotherapy in mCRC patients, with non univocal but generally positive results (Table 1). In the pivotal AVF2107 study, the addition of Bev to the IFL regimen led to a significant increase in PFS (10.6 *vs.* 6.2 months, HR 0.54, *P* < 0.001) and OS (20.3 *vs.* 15.6 months, HR 0.66, *P* < 0.001), independently of *KRAS* mutational status [2]. In the same period a small single-centre randomised trial of Bev added to a similar bolus regimen of irinotecan and 5 fluorouracil showed no difference neither in OS (22.0 *vs.* 25.0 months, *P* = 0.13) nor in the response rate [10].

**Table 1:** First line key trial results with chemotherapy +/− Bevacizumab in mCRC

Chemotherapy	TRIAL (Phase)	No patients	PFS (months)	HR (*P*)	OS (months)	HR (*P*)
IFL [2]	AVF2107 (III)	813	10.6 *vs.* 6.2	0.54 (< 0.001)	20.3 *vs.* 15.6^*^	0.66 (< 0.001)
mFOLFIRI [9]	(III)	222	-	- (−)	22 *vs.* 25^*^	0.13 (−)
FOLFOX/XELOX [11]	NO16966 (III)	1401	9.4 *vs.* 8^*^	0.83 (0.0023)	21.3 *vs.* 19.9	0.89 (0.077)
FOLFOX/FOLFIRI [12]	ITACA (III)	376	9.6 *vs.* 8.4^*^	0.86 (0.182)	20.8 *vs.* 21.3	1.13 (0.304)
Bolus 5FU/AF [14]	(II)	104	9.2 *vs.* 5.0^*^	0.50 (0.0002)	16.6 *vs.* 12.9	0.79 (0.16)
CAPECITABINE [15]	MAX (III)	471	8.5 *vs.* 5.7^*^	0.63 (< 0.001)	18.9 *vs.* 18.9	0.875 (0.314)
CAPECITABINE [16] (elderly patients)	AVEX (III)	280	9.1 *vs.* 5.1^*^	0.53 (<0.0001)	20.7 *vs.* 16.8	0.79 (0.18)

* Primary endpoint of the study

The study NO16966 on Bev added to XELOX or FOLFOX4 revealed a statistically significant improvement in PFS (9.4 *vs.* 8.0 months, HR 0.83, *P* = 0.0023), but not in OS (21.3 *vs.* 19.9 months, HR 0.89, *P* = 0.077) in the overall population [11]. Similarly, the ITACa randomized phase III trial failed to show any benefit from the addition of Bev to first-line standard chemotherapy (FOLFIRI or FOLFOX4). PFS, the primary study endpoint, was 9.6 months for chemotherapy plus Bev and 8.4 months for chemotherapy alone, with a HR of 0.86 (*P* = 0.182). No statistically significant differences in OS or ORR were observed, and results were independent of *KRAS* status [12]. Trials using Bev combined only with fluoropyrimidines reported a significant increase in PFS, but not in OS [13–16].

The head-to-head comparison between anti-VEGF and anti-EGFR agents in combination with first line chemotherapy in KRAS WT mCRC patients was analyzed in 2 large randomized phase III prospective trials, the CALGB/SWOG 80405 and the FIRE-3 [17–19]. A meta analysis of the data from the CALGB/SWOG 80405, the FIRE-3 and the PEAK (a phase II trial comparing FOLFOX-Panitumumab to FOLFOX-B) showed higher ORR and OS with first-line anti-EGFR therapy, compared with anti-VEGF therapy in both KRAS-WT and all RAS-WT patients with mCRC, without any significant impact on PFS [20].

The TRIBE randomized phase III multicenter clinical trial showed that FOLFOXIRI plus Bev significantly improved patients PFS and OS with respect to FOLFIRI plus Bev, and that the efficacy was irrespective of baseline clinical characteristics and *RAS* or *BRAF* mutational status [21].

The vast amount of data from first-line randomized phase III trials supports the use of first-line Bev in combination with chemotherapy in RAS mutated patients (on the basis of superior PFS and OS) and the use of anti-EGFR therapy as an alternative to anti-VEGF therapy in all RAS-WT patients (on the basis of higher ORR and OS rates).

The use of AI in second-line settings is supported by the results of 4 randomized phase III clinical trials, that have been recently undergone (Table 2).

**Table 2:** Second line phase III trial results with chemotherapy +/− Antiangiogenic agents in mCRC

Chemotherapy/Antiangiogenic Agent	TRIAL	No patients	PFS (months)	HR (*P*)	OS (months)	HR (*P*)
FOLFOX4/Bevacizumab [3]	E3200	829	7.3 *vs.* 4.7	0.61 (< 0.0001)	12.9 *vs.* 10.8	0.75 (0.0011)
Chemotherapy switch/Bevacizumab [22]	ML18147	820	5.7 *vs.* 4.1	0.68 (< 0.0001)	11.2 *vs.* 9.8	0.81 (0.0062)
Chemotherapy switch/Bevacizumab [23]	BEBYP	185	6.8 *vs.* 5.0	0.70 (0.010)	14.1 *vs.* 15.5	0.77 (0.043)
FOLFIRI/Aflibercept [4]	VELOUR	1226	6.9 *vs.* 4.67	0.758 (< 0.0001)	13.5 *vs.* 12.1	0.817 (0.0032)

The E3200 was a randomized study on the addition of Bev to second-line FOLFOX4 in patients that were refractory to a fluoropyrimidine/irinotecan-based regimen (not containing Bev) [3]. The FOLFOX4+Bev arm patients had an improved OS (from 10.8 to 12.9 months, *P* = 0.0011) and PFS (7.3 *vs.* 4.7 months, *P* < 0.0001), with respect to those treated with chemotherapy alone.

The ML18147 was a prospective randomized phase III trial assessing the efficacy of Bev continued beyond progression after a first-line Bev containing chemotherapy. The results were in favour of the continuation of Bev, as a higher OS (11.2 *vs.* 9.8 months, HR 0.81, *P =* 0.0062) and PFS (5.7 *vs.* 4.1, HR 0.68 *P* < 0.0001) were shown [22]. The phase III Bevacizumab BeYond Progression (BEBYP) trial was designed similarly to ML18147 and provided confirmatory evidence to support the efficacy of Bev beyond progression [23].

The VELOUR trial was a randomized prospective, placebo controlled, phase III trial investigating the safety and activity of Ziv-aflibercept in combination with second-line FOLFIRI in patients progressing during or after the completion of an oxaliplatin- and a fluoropyrimidine-containing regimen (with or without Bev). An improvement of median OS (13.5 *vs.* 12.1 months, HR 0.817, *P* = 0.0032) and PFS (6.9 *vs.* 4.670 months, HR 0.758, *P* < 0.0001) was reported for patients in the experimental arm rather than in the placebo arm [4].

The results of these trials are similarly indicative of a positive role of AI, also in second-line settings (both in naive patients and in those already treated with Bev in first-line).

The efficacy of regorafenib was evaluated in the randomized, multicenter, placebo controlled CORRECT trial, conducted in mCRC patients refractory to all approved treatment options (fluoropyrimidines, Bev, irinotecan, oxaliplatin and, for *KRAS* WT patients, an EGFR-inhibitor). Patients treated with regorafenib had better OS (6.4 *vs.* 5 months, HR 0.77, *P* = 0.0052) and PFS (HR = 0.49, *P* < 0.0001) rates compared with those in the placebo arm [5].

As not all mCRC patients respond to antiangiogenic agents, numerous trials have been conducted in recent years to find biomarkers capable of identifying a subset of patients who are most likely to benefit from such agents, thus reducing costs and the risk of side-effects. Most of these trials were on Bev, and have been recently reviewed [24]. Unfortunately, several promising biomarkers in preclinical models have failed as predictors of response when tested in clinical trials.

Another crucial point to be considered is that the largest majority of mCRC patients ultimately become resistant to therapy and experience clinical progression. For this reason, the detection of the onset of resistance and the factors that mediate this resistance is becoming increasingly important, in the light of recent data supporting treatment with AI, also beyond progression. Moreover, the pathways of resistance, if identified, could be targeted when tumors become refractory during treatment. In this context hypoxia could play an important role.

## INTERACTION BETWEEN HYPOXIA AND ANGIOGENESIS IN CANCER BIOLOGY

Hypoxia is generally defined as a state of reduced O_2_ availability or decreased O_2_ partial pressures below critical thresholds, that limits or even abolishes the function of organs, tissues, or cells [25]. It represents a stress condition that occurs frequently in several diseases, such as solid tumors [26]. Tumor cells usually respond to hypoxia through activation of several different pathways, leading to a number of biological consequences [27–29]. One of the principal consequences of tumor hypoxia is the induction of the hypoxic inducible factor (HIF) family of transcription factors.

HIF-1 plays a central role in the cellular adaptation to hypoxic conditions. It is a heterodimeric transcription factor consisting of a constitutively active HIF-1β subunit and an oxygen-regulated HIF-1α subunit [30]. In well-oxygenated cells, HIF-1α is subject to O_2_-dependent hydroxylation by prolyl hydroxylase 2 (PHD-2). This event mediates von Hippel-Lindau tumor suppression protein (pVHL) binding and subsequent ubiquitination and proteasomal degradation [31, 32]. Under hypoxia conditions, HIF-1α is stabilized and it dimerizes with HIF-1β. The complex moves to the nucleus where it binds to hypoxia response elements (HREs) within regulatory regions of target genes [33, 34]. The heterodimeric protein can regulate the expression of numerous genes involved in angiogenesis, erythropoiesis, proliferation, glycolytic tumor metabolism, metastasis, autophagy, apoptosis and pH regulation [35, 36].

Hypoxia and HIF pathway activation in tumor cells are important stimuli for blood vessel growth. Tumor growth indeed depends on the development of tumor neovasculature [37]. Tumor angiogenesis can be defined as the formation of new blood vessels from pre-existing vessels in a process known as sprouting angiogenesis [38]. Colorectal cancer represents a model for investigating the effects of angiogenesis throughout tumorIt has high angiogenesis scores and microvessel densities, that are associated with a high risk of metastases, recurrence, and early patient death [39]. ngiogenesis plays a crucial role in CRC growth, proliferation and metastatization and it has been investigated as a potential target for treatment of metastatic disease [40, 41]. Angiogenic properties, in particular the balance of angiogenic activators and inhibitors, are necessary for this cancer to growth and survive, and thus to develop from a quiescent tumor into a more aggressive tumor. This transition is called angiogenic switch [42, 43].

HIF-1 can directly activate the expression of a number of pro-angiogenic factors, including vascular endothelial growth factor (VEGF), VEGF receptors, plasminogen activator inhibitor-1 (PAI-1), angiopoietins (Ang-1 and -2), platelet-derived growth factor β (PDGF-β), the Tie-2 receptor, and matrix metalloproteinases (MMP-2 and -9) [44–46]. An essential mediator of angiogenesis is VEGF family, which includes 5 glicoproteins (VEGF-A, VEGF-B, VEGF-C, VEGF-D, and placental growth factor [PlGF]), 3 receptors (VEGFR-1/Flt-1, VEGFR-2/Flk-1/KDR, and VEGFR-3/Flt-4), and 2 co-receptors (neuropilin NP1 and NP2) [47–50]. VEGFR-2 is the central mediator of VEGF-stimulated tumor angiogenesis [47, 51]. When VEGF-A binds to VEGFR-2, this receptor become phosphorylated, and signalling pathways like the Ras-Raf-MAPK, and the PI3K-AKT pathways are activated resulting in endothelial cell migration, proliferation and survival, and tube formation [52, 53].

After binding to VEGF-A, VEGF-B and PlGF, VEGFR-1 activation appears to be crucial in the epithelial-to-mesenchymal transition [54]. VEGFR-3 binds to VEGF-C and -D and is involved in lymphangiogenesis [55]. PlGF promotes the survival of endothelial cells and can modulate the effects of VEGF-A on angiogenesis [56]. Different pathways and molecules involved in angiogenesis have been targeted for the treatment of colorectal cancer [57] (Figure 1).

VEGF has been shown to be involved in the growth and development of colorectal cancer. In this type of tumor, VEGF expression can correlate with invasiveness, vascular density, metastasis, recurrence, and prognosis [13, 40, 58, 59].

HIF-1 also regulates induces the expression of angiopoietins (Ang-1 and Ang-2) and their receptor Tie-2. Ang-1 and Ang-2 are specific ligands of Tie-2: its activation promotes endothelial cell survival and vascular maturation. Ang-1 induces vessel stabilization, while Ang-2 is an antagonist of Ang-1 and mediates vascular destabilization. Ang-2 is expressed in the vasculature of many tumors, and may act synergistically with VEGF to promote tumor-associated angiogenesis and tumor progression [60].

Besides HIFs, other important adaptive mechanisms to hypoxia are the unfolded protein response (UPR) and the induction of AKT-mTOR signaling pathway [61].

Under hypoxia, incorrectly folded proteins can accumulate in the endoplasmic reticulum, disrupting cellular integrity. UPR increases degradation of unfolded proteins and restores homeostasis reducing endoplasmic reticulum stress [62, 63].

HIF-, UPR-, and mTOR-dependent responses to hypoxia serve to tumor cells to survive these stress conditions and seem to act in an integrated way, influencing common downstream pathways affecting gene expression, angiogenesis, metabolism, cell survival, tumorigenesis, and tumor growth [61].

In summary, hypoxia is the prime driving force for tumor angiogenesis, where imbalance of pro-angiogenic and anti-angiogenic factors occurs [38].

## THE ROLE OF HYPOXIA IN ACQUIRED RESISTANCE TO ANTI-ANGIOGENETIC TREATMENT

As angiogenesis is an adaptive response to tissue hypoxia [64], a vicious cycle between hypoxia and angiogenesis can be observed in tumors. In most malignant tumors there is an imbalance between the supply and consumption of oxygen that generates hypoxic regions. This condition stimulates the production of pro-angiogenic factors leading to tumor growth with aberrant and chaotic vasculature. In turn, the morphological and functional deformed blood vessels lead to a heterogeneous distribution of oxygen, diminishing oxygen delivery to tumor cells [65].

Hypoxia can have negative implications for clinical outcome. It can enhance colon cancer migration and invasion through promotion of epithelial-mesenchymal transition (EMT) process [66, 67], and lead to therapeutic resistance [68–70].

**Figure 2:**
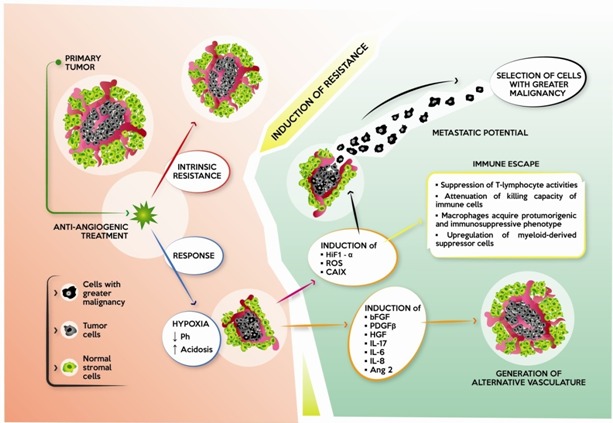
Effect of anti-angiogenic therapy on cancer Some tumors are intrinsically resistant to anti-angiogenic drugs, while others initially respond with a reduction in tumor mass and vasculature, and induction of hypoxia. Hypoxia lowers pH and creates a condition of acidosis, with the subsequent development of mechanisms of resistance: 1) induction of alternative pro-angiogenic factors, *e.g.* bFGF, PDGF-β, HGF, IL-17, IL-6, IL-8 and Ang 2, which restore tumor vasculature; 2) induction of HIF-1α, which, in turn, induces the transcription of other factors, increasing metastatic potential and invasiveness of tumor cells. Tumor cells with greater malignancy are selected; and 3) immunomodulation through different mechanisms leading to immune escape.

During treatment with an anti-angiogenic drug, tumors develop several strategies to evade the anti-angiogenic effect. While some tumors are intrinsically refractory and fail to respond to such drugs even at early stages of treatment, others acquire evasive resistance mechanisms to circumvent angiogenic blockade. These mechanisms can involve the secretion of alternative pro-angiogenic mediators such as bFGF, PDGF-β, IL-17, IL-6, IL-8, Ang-2 and HGF, which may reactivate the revascularization program [71]. Another mechanism of resistance induction is hypoxia (Figure 2). Although anti-angiogenic drugs act by blocking blood supply to the tumor, the generation of an hypoxic condition may fuel tumor progression and treatment resistance. This represents a paradoxical effect concerning the activity of anti-angiogenic drugs, inhibiting nutrient supply to the tumor but at the same time inducing an increase in its metastatic potential and invasiveness.

Hypoxia within tumor increases during treatment with an anti-angiogenic agent, inducing pH drop and consequent acidosis [66]. Several reports in the literature have demonstrated a straight correlation between hypoxia and the increase of metastatic potential, suggesting several hypotheses to explain this [67, 68, 72, 73]. Hypoxia condition activates the transcription factor HIF-1α, that induces activation of p53 and cyclin-dependent kinase inhibitors [74]. HIF-1α also induces the expression of carbonic anhydrase IX, that promotes cell survival and invasion [75]. Moreover, the production of reactive oxygen species (ROS) can promote cell motility [72] and create a favorable condition for gene mutation and genomic instability [76] (Figure 2).

Although increased metastasis with anti-angiogenic therapy has been found in pre-clinical models, extensive reviews of patterns of relapse on and off Bev do not support this pre-clinical observation [77]. One possible explanation for this is the effect of additional chemotherapeutic drugs combined with anti-angiogenics in clinical practice such as doxorubicin, topotecan and gemcitabine, that counteract the sunitinib-induced metastatic dissemination of the Lewis lung carcinoma xenograft models [78].

Another mechanism through which hypoxia promotes cancer invasion is mediated by immune modulation (Figure 2). Under physiological conditions, immune cells control the normal destruction of pathogens, foreign antigens and abnormal cells. Conversely, in a hypoxic microenvironment, macrophages, that usually recognize, engulf and remove dying cells, are converted into cells with a protumorigenic and immunosuppressive phenotype [79–85]. Lactic acid produced by tumor cells, as a by-product of aerobic or anaerobic glycolysis, is critical for signaling, as it induces the expression of vascular endothelial growth factor and the M2-like polarization of tumor-associated macrophages. This effect is mediated by HIF-1α. Moreover, the lactate-induced expression of arginase 1 by macrophages is pivotal in tumor growth [80]. Similarly, hypoxia and acidosis attenuate the killing capacity of immune effector cells in tumor microenvironment. Specific hypoxia-induced growth factors and cytokines, such as transforming growth factor beta (TGF-β) and VEGF are able to suppress the activity of T lymphocytes and the ability of dendritic cells to process antigens, and present them to lymphocytes. Another mechanism through which hypoxia can suppress the immune system is the up-regulation in myeloid-derived suppressor cells, dendritic cells and cancer, of the immune checkpoint protein PD-L1, *via* HIF-1α [86]. This favors immune suppression and evasion.

Hypoxic conditions could also select more malignant cells, i.e. those able to grow in hypoxic conditions [87] (Figure 2). It produces a pressure mechanism that selects tumor cells with increased aggressiveness and lower sensitivity to anti-angiogenic therapy [88, 89].

All these aspects indicate that hypoxia, inevitably induced during treatment with anti-angiogenic drugs, may, in turn, represent a resistant mechanism to drug activity. Mesange *et al* concluded that resistance to Bev in preclinical models of colorectal cancer may be due to an upregulation of the autocrine HIF-VEGF-VEGFR signaling and to increased tolerance to hypoxia, probably caused by prolonged exposure to Bev [90]. Interestingly, the authors demonstrated that although these resistance mechanisms were related to Bev, they did not influence sensitivity to other anti-angiogenic drugs, such as nintedanib. Moreover, a recent report on colorectal cancer xenograft models showed that Bev induced important metabolic modifications within the tumor due to alterations in the expression of proteins involved in glucose, lipid and fatty acid metabolism (*e.g.* GPD2, ATP5B, STAT3, FASN) and in hypoxic regulation and vasculogenesis (*e.g.* ATP5B, THBS1, HSPG2) [91].

Jain *et al* have recently advanced a theory on how a judicious use of anti-angiogenic agents could transiently “normalize” the abnormal tumor vasculature, resulting in improved blood perfusion [66]. This should decrease hypoxia and increase drug accessibility. He hypothesized that therapies administrated during the window of normalization might achieve greater efficacy. On the other hand, high doses of anti-VEGF/R agents could cause a rapid reduction in blood perfusion with consequent hypoxia, resulting in increased metastatic potential and resistance to drugs [66]. No clinical data directly comparing the dose effect of anti-VEGF agents on perfusion or oxygen levels are present in the literature.

However, several studies have reported that Bev treatment results in vascular normalization. A study on colorectal cancer patients with liver metastases given neoadjuvant chemotherapy and Bev showed that treatment resulted in tumor vessel stabilization, leading to more mature, stable vessels with an increased diameter, whilst also decreasing vascular density and increasing necrosis [92].

## MONITORING HYPOXIA DURING ANTIANGIOGENIC TREATMENTS COULD HAVE A PREDICTIVE ROLE

The three main vascular responses to anti-angiogenic therapy have already been described using Magnetic Resonance Imaging (MRI): reduced perfusion, no perfusion response and increased perfusion [93–95]. Some studies have used MRI as a method to evaluate patient response to anti-angiogenic therapy in different solid tumors including colorectal cancer [93, 95–99].

Likewise, in another study a rapid decrease in tumor perfusion was observed after Bev, and associated with a decrease of VEGF expression [94]. As the observed decrease in perfusion in tumors was probably too rapid to be solely ascribed to inhibition of tumor angiogenesis, vasoconstrictive effects of anti-angiogenic drugs on tumor vessels, particularly those from the host, should be considered as a potential underlying mechanism. In this regard, inhibition of endothelial nitric oxide synthesis by VEGF inhibitors may be considered an important factor. In this context, our recent results have demonstrated a significant association of specific endothelial nitric oxide synthase gene polymorphisms with response, PFS and OS of mCRC patients treated with Bev-based chemotherapy [100], suggesting a possible connection between the activity of this protein and the antiangiogenic drug efficacy.

Other studies reported vascular normalization at the start of therapy in about half of the patients, whereas the other half reported reduced perfusion or no perfusion response [95, 101]. Moreover, a rise in survival effects was identified in patients who showed a higher tumor perfusion after anti-angiogenic therapy, due to increased chemotherapeutic delivery [95]. In particular, the authors of this study developed a technique called vessel architectural imaging (VAI) to measure ΔSO_2_ (the fraction saturation of hemoglobin with oxygen), that is a parameter sensitive to changes in blood oxygenation reflecting the relative difference between arteriole and venule oxygen saturation levels, and, as a consequence, tissue oxygen consumption. By using this parameter they demonstrated that patients responding to cediranib (an anti-angiogenic drug) were those with increased perfusion and higher delivery of oxygen to the tumor [95].

Additional clinical study should be performed to clarify the role of tumor perfusion in monitoring response to anti-angiogenic agents.

Another way to monitor hypoxia drug response is to use circulating biomarkers.

The biological link between hypoxia, lactate dehydrogenase (LDH) levels and the tumor-driven angiogenesis pathway through the abnormal activation of HIF-1α is well established. HIF-1α is an important transcription factor that upregulates a series of genes involved in glycolytic metabolism, angiogenesis, cell survival and erythropoiesis. Among others, HIF-1α also regulates activates transcription of several glycolytic enzymes, such as LDH [102]. As LDH and pro-angiogenesis factors are regulated by the same HIF-1α-driven molecular pathway, high LDH levels are concomitantly present along with abnormal activation of the VEGF pathway [103]. Accordingly, Azuma *et al* demonstrated that high LDH serum levels were associated with tumor overexpression of VEGF-A and VEGFR-1 in mCRC patients [104]. It has also been speculated that LDH levels may represent an indirect indicator of activated tumor angiogenesis and of worse prognosis [105, 106]. A study by Scartozzi *et al* suggested that mCRC patients treated with Bev and showing high pre-treatment LDH serum levels experienced an improved probability of response and an equivalent median PFS and OS when compared with patients with lower LDH serum levels [107]. Moreover, together with other research teams we demonstrated that mCRC patients with high LDH levels have a poor prognosis, and that the addition of Bev seems to improve prognosis of this group of patients, assimilating it to that of patients with low LDH levels [108, 109]. Similarly, Bar *et al* showed that high total serum LDH correlated with shorter PFS, and high hypoxia-related LDH isoenzymes correlated with worse PFS and OS, in patients treated with either cediranib or Bev [110].

Other preliminary results from our laboratory showed that high pretreatment level of HIF-1α were associated with a low response rate in mCRC patients treated with Bev-based chemotherapy [111].

## RATIONALE FOR COMBINATION TREATMENT STRATEGIES

Given that hypoxia seems to represent a resistance mechanism to anti-angiogenic treatment, combination strategies using a concomitant treatment with anti-angiogenic drugs and inhibitors of hypoxia could be promising.

Results obtained in first-line treatment of mCRC have shown significant results using combination strategies with Bev and chemotherapeutic regimens containing irinotecan [2]. The synergism observed with this combination approach may be attributed also to the ability of camptothecin to inhibit HIF-1α [112], reducing part of the resistance mechanisms to Bev. Accordingly, a completed phase I clinical trial on different solid tumors combined Bev with the camptothecin analogue EZN-2208 (PEGylated SN38). The authors reported a reduction in HIF-1α protein levels in tumor biopsies compared to baseline in 5 of 7 patients considered [113]. Similarly, a previous *in vitro* study demonstrated that the combination of Bev with topotecan, another camptothecin that inhibits HIF-1α translation through DNA damage-independent mechanisms [114], had a more remarkable therapeutic effect compared to the single drugs alone in a xenograft model of glioblastoma [115]. The authors reported a strong tumor proliferation inhibition and induction of apoptosis, and highlighted the potentiality of this combination approach, suggesting further investigation of this therapeutic strategy in future clinical trials.

A number of ongoing clinical trials are evaluating the effects of these combination approaches (ClinicalTrials.gov).

A recent phase I trial was carried out to study the combining effect of Bev plus bortezomib, an agent that suppresses HIF-1α transcriptional activity, in patients with different solid tumors [116]. The study showed that the treatment was well-tolerated and that 12% of patients had a clinical benefit (partial response or stable disease for more than 6 months).

One phase I clinical trial is studying the effect of Bev in combination with the mTOR inhibitor MLN0128 (NCT02142803) in glioblastoma and other solid tumors. Targeting mTOR has a twofold effect, as it upregulates HIF expression and is an independent metabolism regulator [117].

There are 4 phase I/II clinical trials combining the hypoxia activated prodrug TH-302, an alkylating agent with an anti-angiogenic therapy in a variety of tumors (NCT01497444, NCT01381822, NCT01403610, NCT01485042) [118]. TH-302 enhances the activity of a wide range of conventional anti-neoplastic agents in a broad panel of *in vivo* xenograft models, including colorectal cancer models [118].

More recently, salternamide A was identified as an inhibitor of HIF-1α accumulation under hypoxic conditions in cancer cells and was shown to have an anti-proliferative effect on HCT116 colorectal cancer cells. Thus, it could be a leading candidate for the development of anticancer agents and could be considered for use in combination with an AI [119].

The synergistic effect observed with the use of chemotherapy in combination with Bev could also be attributable to the fact that chemotherapy itself is a potent anti-angiogenic agent, and Bev, by blocking VEGF activity, could inhibit the resistance-response induced by the chemotherapeutic agent, sensitizing both endothelial and cancer cells to therapy [120].

## CONCLUSIONS

Anti-angiogenic therapy is based on the concept that angiogenesis is required for tumor growth: destruction of the tumor vasculature would deprive the tumor of oxygen and nutrients, inducing cell growth inhibition. However, as tumor vasculature is structurally abnormal and functionally inefficient, the resultant hypoxic microenvironment is associated with tumor progression and resistance to therapies. Therapeutic destruction of the tumor vasculature is expected to yield more severe hypoxia. Hypoxia, in turn, is able to induce additional angiogenic responses through the activation of HIF-1α, to drive genetic alteration responsible for malignant progression, and to modulate the immune system inducing immune-escape.

AI in combination with chemotherapy are the standard of care in mCRC patients. Unfortunately, there are no biomarkers that can be used as predictor of response and resistance.

The detection of the onset of resistance to AI is becoming increasingly important, in the light of recent data supporting treatment with AI even beyond progression. In this context, hypoxia could play an important role, as mentioned before.

Combination strategies of AI with agents able to inhibit hypoxia could be very promising. In mCRC, the efficacy demonstrated with the combination of AI and irinotecan-based chemotherapy could be also attributed to the capacity of camptothecin to inhibit HIF-1α. This strategy could improve the efficacy of anti-angiogenic treatments in mCRC and in other types of cancer.

In summary, hypoxia may represent an important parameter to assess during the course of anti-angiogenic treatment, and it could be useful in monitoring response and determining resistance. Further studies are needed to define its role in the definition of combination strategies of therapy.
